# Mortality burden attributable to long-term exposure to fine particulate matter among older adults in Korea

**DOI:** 10.4178/epih.e2025028

**Published:** 2025-05-28

**Authors:** Jongmin Oh, Jisun Myung, Changwoo Han, Hyun-Joo Bae, Soontae Kim, Yun-Chul Hong, Dong-Wook Lee, Youn-Hee Lim

**Affiliations:** 1 Department of Environmental Medicine, Ewha Womans University College of Medicine, Seoul, Korea; 2 Institute of Ewha-SCL for Environmental Health (IESEH), Ewha Womans University College of Medicine, Seoul, Korea; 3 Department of Human Systems Medicine, Seoul National University College of Medicine, Seoul, Korea; 4 Inha Research Institute for Medical Science, Inha University College of Medicine, Incheon, Korea; 5 Department of Preventive Medicine, Chungnam National University College of Medicine, Daejeon, Korea; 6 Division of Environmental Health, Korea Environment Institute, Sejong, Korea; 7 Department of Environmental and Safety Engineering, Ajou University, Suwon, Korea; 8 Institute of Environmental Medicine, Medical Research Center, Seoul National University, Seoul, Korea; 9 Department of Occupational and Environmental Medicine, Inha University Hospital, Inha University, Incheon, Korea; 10 Section of Environmental Health, Department of Public Health, University of Copenhagen, Copenhagen, Denmark

**Keywords:** Cohort studies, Particulate matter, Aged, Environmental exposure, Health impact assessment

## Abstract

**OBJECTIVES:**

This study aimed to evaluate the association between long-term exposure to particulate matter with an aerodynamic diameter <2.5 μm (PM_2.5_) and cause-specific mortality among older adults in Korea, providing insights into the evolving public health burden in an aging society.

**METHODS:**

We analyzed national insurance claims data spanning 2010-2019. Modeled PM_2.5_ concentrations were assigned to participants according to their residential districts. We employed time-varying Cox proportional hazard models, using age as the time scale, adjusted for potential confounders. Six cause-specific mortalities were considered: ischemic heart disease (IHD), stroke, chronic obstructive pulmonary disease (COPD), acute lower respiratory infection (ALRI), lung cancer (LC), and type 2 diabetes mellitus (T2DM). Annual excess deaths attributable to long-term PM_2.5_ exposure were calculated.

**RESULTS:**

A total of 5,360,032 older adults were followed from 2010 to 2019. Hazard ratios (HRs) per 10 μg/m^3^ increase in 12-month PM_2.5_ concentration were as follows: IHD, 1.068 (95% CI, 1.040 to 1.097); stroke, 1.023 (95% CI, 1.003 to 1.043); ALRI, 1.050 (95% CI, 1.026 to 1.076); COPD, 1.114 (95% CI, 1.072 to 1.157); T2DM, 1.046 (95% CI, 1.007 to 1.086); and LC, 0.972 (95% CI, 0.948 to 0.996). Excess deaths attributable to long-term PM_2.5_ exposure were estimated at 4,888 (95% CI, 2,304 to 7,323) in 2010 and 5,179 (95% CI, 2,585 to 7,648) in 2019.

**CONCLUSIONS:**

Although PM_2.5_ levels in Korea have shown a declining trend over the past decade, mortality among older adults associated with long-term PM_2.5_ exposure has not significantly decreased, likely due to the rapid aging of the population.

## GRAPHICAL ABSTRACT

[Fig f3-epih-47-e2025028]

## Key Message

A retrospective cohort study of 5.36 million Korean older adults (2010-2019) found that long-term exposure to PM_2.5_ was associated with increased mortality from ischemic heart disease, stroke, ALRI, COPD, and type 2 diabetes mellitus. Despite improved air quality, excess deaths attributable to PM_2.5_ increased from 5,241 in 2010 to 5,696 in 2019, driven by rapid population aging. Population aging continues to offset gains from air quality improvements, highlighting the persistent public health burden of PM_2.5_ among older adults.

## INTRODUCTION

The long-term and cumulative health effects of particulate matter with an aerodynamic diameter <2.5 μm (PM_2.5_) on all-cause and cause-specific mortality have been consistently documented [1-3], including a systematic review and a meta-analysis [4]. According to a 2019 report by the World Health Organization (WHO), over 99% of the global population is exposed to air pollution levels exceeding WHO air quality guidelines, resulting in approximately 6.67 million deaths annually [5]. Among these deaths, exposure to ambient PM_2.5_ accounts for the largest proportion. Major cause-specific mortalities linked to long-term PM_2.5_ exposure include ischemic heart disease (IHD), stroke, acute lower respiratory infections (ALRI), chronic obstructive pulmonary disease (COPD), lung cancer (LC), and type 2 diabetes mellitus (T2DM) [4-7].

According to the 2023 United Nations report, in 2021, 1 in every 10 people worldwide was aged 65 or older; by 2050, this proportion is expected to increase to 1 in 6 [8]. The countries with the oldest populations are shifting geographically from Europe toward Eastern and Southeastern Asia. Notably, Korea’s population of older adults is anticipated to comprise approximately 40% of the national population by 2050.

Older adults are particularly susceptible to adverse health impacts from PM_2.5_ exposure [3,9-12]. Although PM_2.5_ concentrations in Korea have gradually decreased [13], the rapidly growing population of older adults may maintain or even increase the mortality burden attributable to long-term PM_2.5_ exposure. This demographic shift toward an aging population necessitates research focused specifically on the mortality burden among older adults [14].

The mortality burden attributable to PM_2.5_ can be estimated using 4 key factors: population at risk, baseline mortality rates, exposure levels, and the exposure–response function between PM_2.5_ and mortality [6,7]. Although researchers have generally reached a consensus on the first 3 factors, exposure–response functions have varied among studies. Numerous health impact assessment studies have utilized either the integrated exposure-response function [7], the Global Exposure Mortality Model [6], or pooled estimates from meta-analyses [4,15]. However, these exposure–response functions predominantly originate from Western cohort studies conducted among populations exposed to relatively lower air pollution levels (e.g., in the United States, Europe, and Canada) [4,6,7]. While these exposure–response functions can provide useful global estimates, their applicability to specific regions such as Asia may be limited due to potential differences in ethnicity, cultural practices, and exposure distributions.

Therefore, to obtain a more accurate estimation of the mortality burden attributable to PM_2.5_ exposure among older adults, we utilized an exposure–response function derived from nationwide population-based cohort data and exposure modeling specific to Korea. This study aimed to evaluate the association between long-term PM_2.5_ exposure and 6 cause-specific mortalities (IHD, stroke, ALRI, COPD, LC, and T2DM) and to estimate the annual excess deaths attributable to long-term PM_2.5_ exposure among older Koreans, considering the ongoing rapid demographic aging in the country.

## MATERIALS AND METHODS

### Study population

We targeted 5,562,993 adults aged 65 years and older in 2010. To construct a retrospective cohort of senior adults, we utilized demographic variables, health insurance claim data, and vital status from the National Health Information Database (NHID; NHIS-2021-1-694). Because health insurance coverage is mandatory for individuals legally residing in Korea, NHID provides comprehensive data for almost the entire population [16]. NHID data span national, provincial, and municipal levels, effectively covering nearly all residents in Korea [17]. NHID records, linked to death registry data provided by Statistics Korea, were used to identify causes of death among older adults. Among older adults in 2010, we excluded subjects with missing demographic information, such as gender, age, income level, and area of residence (n=201,804, 3.63%), as well as subjects with death records prior to 2010 (n=1,157, 0.02%). To avoid bias introduced by increased mortality during the coronavirus disease 2019 pandemic, the study period was restricted to January 2010 through December 2019. During the follow-up period, deaths from causes not of interest in our analysis were censored.

### Outcome definition

We defined the major causes of death related to long-term exposure to PM_2.5_ as primary health outcomes. Causes of death were selected based on previous studies demonstrating associations with PM_2.5_ exposure [3,4,6,7,11,12,18-21] (Supplementary Material 1). Data on the selected causes of death and its International Classification of Diseases 10th revision (ICD-10) codes are as follows: IHD (I20-I25), stroke (I60-I69), ALRI (J12-J18, J20-J22), COPD (J40-J44), LC (C33-C34), and T2DM (E11).

### Particulate matter with an aerodynamic diameter <2.5 μm data

The PM_2.5_ exposure data for administrative districts were predicted using the Community Multiscale Air Quality model (CMAQ, version 4.7.1), with a spatial resolution of 9 km×9 km. Since nationwide PM_2.5_ monitoring in Korea was not implemented before 2015, we relied on modeled PM_2.5_ data. Modeled PM_2.5_ concentrations employed in this study have been widely used in environmental epidemiological studies conducted in Korea [3,22-24]. The CMAQ model demonstrated high correlation with monitored data from 2010 to 2019 (Supplementary Material 2). Monthly correlations between monitored and modeled PM_2.5_ concentrations across cities and provinces ranged from 0.878 to 0.999 during 2015-2019. A detailed description of the CMAQ model is provided in Supplementary Material 3 [3,22-24]. We linked 12-month moving average PM_2.5_ exposure values to individuals based on their residential district (250 administrative districts). Because residential information was updated annually, exposure assignments were revised each calendar year to account for residential relocation.

### Covariates

Individual-level covariates included gender, income level at baseline, insurance enrollment type (self-employed or employee subscriber), area of residence (7 metropolitan cities and 9 provinces), and underlying diseases at baseline (2010). Income levels, segmented into 20 quantiles, were categorized into 6 groups: Medical Aid recipients (category 0), very low (1-4), low (5-8), moderate (9-12), high (13-16), and very high (17-20). The Charlson comorbidity index (CCI) was calculated using health insurance claims data from NHID for the year preceding the baseline (January-December 2009). Underlying diseases were determined based on the CCI, with a CCI score of 1 or higher classified as “yes,” and a score of 0 classified as “no.”

In this study, individual-level potential confounders such as smoking, alcohol consumption, and physical activity could not be directly adjusted due to their absence in claims data. Therefore, we used district-level confounders, as suggested in previous studies. We obtained the population, percentage of the population aged 65 years and older (%), education level (i.e., the proportion of individuals with high school diplomas [%]), and smoking rate (%) from the Korean Statistical Information Service. The annual mean temperature (°C) and rainfall (mm) were obtained from the Korea Meteorological Administration. All district level variables were adjusted as time-varying covariates to reflect temporal changes (i.e., yearly). For the proportion of older adults with high school diplomas, data were collected at 5-year intervals in 2010 and 2015. Therefore, 2010 data were assigned for the period 2010-2014, and 2015 data were assigned for 2015-2019.

### Associations between long-term exposure to particulate matter with an aerodynamic diameter <2.5 μm and cause-specific mortality

To evaluate the association between long-term exposure to PM_2.5_ and cause-specific mortality, we employed a time-varying Cox proportional hazard model. The time variable was defined in monthly intervals (from 1 to 120 months). Biological age was used as the time scale to examine age-dependent associations between long-term PM_2.5_ exposure and cause-specific mortality [25]. We constructed 4 models: Model 1: unadjusted; Model 2: adjusted for gender, type of insurance enrollment, income level, calendar year, and residence area (using strata terms); Model 3: adjusted for all covariates from model 2 and underlying diseases; Model 4: adjusted for all covariates from model 3 and district-level confounders. The model with the lowest Akaike information criterion (model 4) was selected as the main model (Supplementary Material 4).

To investigate the exposure-response relationship between the 12-month moving average PM_2.5_ and cause-specific mortality risk, we tested for non-linearity using restricted cubic spline models with varying knots (3, 4, and 5). Among these models, the spline with 3 knots had the lowest Akaike information criterion and was thus selected.

We performed subgroup analyses by gender, age group (young-old [65-74 years] vs. old-old [≥75 years]) [26], and Medical Aid status (Medical Aid beneficiaries vs. non-Medical Aid beneficiaries). A t-test was conducted to assess differences in effect size (log hazard ratio [HR]) between the 2 groups in each subgroup analysis, calculated as follows:


$$t=\frac{\hat{\beta_{1}}-\hat{\beta_{2}}}{SE\left(\hat{\beta_{1}}-\hat{\beta_{2}}\right)}$$

where *β* indicates the effect size, and SE indicates the standard error for each effect size.

Four sensitivity analyses were conducted. First, we analyzed the association between 24-month, 36-month, and 48-month moving average PM_2.5_ exposure and cause-specific mortality. Second, we performed the same analysis after excluding individuals who died within 1 month, 2 months, and 3 months from the baseline. Third, we performed the analysis on individuals who did not move between the 16 regions during the study period. Fourth, we constructed a two-pollutant model by adjusting for sulfur dioxide (SO_2_), nitrogen dioxide (NO_2_), and ozone (O_3_) to estimate the effect size of PM_2.5_. All estimated HRs with 95% confidence intervals (CIs) were presented per 10 μg/m^3^ increase in PM_2.5_ concentration.

### Mortality burden attributable to particulate matter with an aerodynamic diameter <2.5 μm

We estimated the mortality burden attributable to long-term exposure to PM_2.5_ for 5 specific causes (IHD, stroke, ALRI, COPD, and T2DM) from 2010 to 2019. Because our study did not identify a positive association for LC, we did not estimate excess deaths for LC. Excess mortality rates per 100,000 older adults were also calculated to facilitate comparisons across specific causes. Excess deaths were estimated using the equation:


$$\text{ Excess death }=\text{ Deaths }\times \left(1-\frac{1}{\exp\left(\beta \times \Delta {\mathrm{PM}}_{2.5}\right)}\right)$$

where “Deaths” represents the number of deaths due to a specific cause among individuals aged 65 years or older, *β* indicates the log HR from the main model (model 4) in this study. ΔPM_2.5_ indicates the difference in the concentration of PM_2.5_ between the current level and a reference level, which is assumed to be the baseline concentration for comparison. In other words, if the current concentration is higher than the reference level, the difference represents the health impact that can be attributed to that level of pollution. We set the reference concentration to 5 μg/m^3^ according to the 2021 WHO air quality guideline [27]. Excess deaths were computed using annual mortality and PM_2.5_ concentration data. The 95% CIs for excess deaths were based on the 95% lower and upper bounds of the HR associated with changes in PM_2.5_ concentrations. Excess mortality rates were standardized per 100,000 population. Our estimates were also compared to mortality burdens reported in recent systematic reviews [4]. Since no significant associations were observed for LC and T2DM, excess mortality was estimated only for IHD, stroke, ALRI, and COPD.

Data preprocessing, statistical analyses, and visualization were conducted using SAS version 9.4 (SAS Institute Inc., Cary, NC, USA) and R version 4.2.1 (R Foundation for Statistical Computing, Vienna, Austria). Statistical significance was set at a p-value <0.05.

### Ethics statement

This study was exempt from review by the Institutional Review Board of Seoul National University Hospital, Korea (IRB No. E-2105-043-1218). Since personal information managed by NHIS was not accessed by the authors, ethical approval was not required.

## RESULTS

In total, 5,360,032 older adults were analyzed in this study (Supplementary Material 5). The mean±standard deviation (SD) age of older adults was 73.4±6.5 years. Demographic characteristics of the study population are shown in Table 1. Of the total, 62.9% belonged to the young-old group (65-74 years), and 37.1% to the old-old group (≥75 years). Men accounted for 40.9% of participants, while women comprised 59.1%. Approximately 40.9% lived in the 7 metropolitan cities, and 59.1% resided in the 9 provinces. Around 60.5% of older adults had underlying diseases. From 2010 to 2019, the districts’ mean± SD population size, proportion of older adults (%), proportion with high school diplomas (%), smoking rate (%), annual mean temperature (°C), and rainfall (mm) were 205,339.0±161,903.5, 17.5±7.9, 37.1±14.0, 23.1±3.1, 12.5±1.4, and 1,262.0±366.6, respectively (Supplementary Material 6).

**Figure 1.**
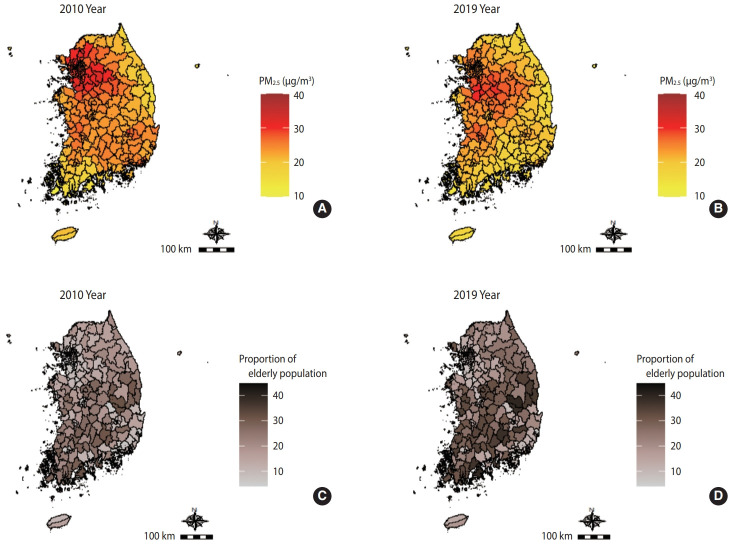
Map of the distribution of average particulate matter with an aerodynamic diameter <2.5 μm (PM_2.5_) concentration in (A) 2010 and (B) 2019 and proportion of older adults in the population aged 65 and over in (C) 2010 and (D) 2019 by district in Korea.

During the follow-up period from 2010 to 2019 (person-years: 44,936,374), 1,774,563 older adults died of any cause. Among these deaths, 5.6% (n=99,617) died due to IHD, 10.2% (n=180,930) due to stroke, 7.0% (n=124,022) due to ALRI, 2.6% (n=46,228) due to COPD, 6.2% (n=110,520) due to LC, and 2.7% (n=47,707) due to T2DM (Table 2). The mean event time by cause of death ranged from a minimum of 54.0 months (T2DM) to a maximum of 72.4 months (ALRI) (Table 2). Figure 1 illustrates the concentrations of PM_2.5_ and the proportion of older adults in the population in 2010 and 2019. Over the 10-year period, the PM_2.5_ concentration decreased, whereas the proportion of older adults increased.

Table 3 presents the associations between 12-month moving average PM_2.5_ exposure and cause-specific mortality. In the fully adjusted model (model 4), HRs per 10 μg/m³ increase in PM_2.5_ were 1.068 (95% CI, 1.040 to 1.097) for IHD, 1.023 (95% CI, 1.003 to 1.043) for stroke, 1.050 (95% CI, 1.026 to 1.076) for ALRI, 1.114 (95% CI, 1.072 to 1.157) for COPD, and 1.046 (95% CI, 1.007 to 1.086) for T2DM. In contrast, no significant association was observed between 12-month moving average PM_2.5_ exposure and LC mortality.

Supplementary Material 7 displays the non-linear exposure-response associations between long-term PM_2.5_ exposure and mortality from IHD, stroke, COPD, and T2DM. Mortality risks for IHD and stroke increased linearly at lower concentrations but decreased at higher concentrations. For COPD mortality, risk continuously increased across the PM_2.5_ range, while T2DM mortality exhibited a U-shaped pattern. Additionally, we found a linear association between PM_2.5_ and ALRI mortality.

Supplementary Material 8 presents subgroup analyses by gender. Positive associations between 12-month PM_2.5_ exposure and mortality from IHD and T2DM were found in women, whereas no significant associations were observed for men. Stroke mortality did not differ significantly by gender. For COPD mortality, HR was slightly higher in women (HR, 1.163; 95% CI, 1.088 to 1.244) compared to men (HR, 1.095; 95% CI, 1.046 to 1.147).

Supplementary Material 9 shows subgroup analyses by age group (young-old [65-74 years] vs. old-old [≥75 years]). For IHD, stroke, COPD, and T2DM mortality, positive associations were observed primarily in the old-old group (≥75 years). In contrast, ALRI mortality showed a slightly higher HR in the young-old group (HR, 1.121; 95% CI, 1.026 to 1.225) than in the old-old group (HR, 1.045; 95% CI, 1.019 to 1.071).

Supplementary Material 10 shows subgroup analyses by Medical Aid status (Medical Aid beneficiaries vs. non-Medical Aid beneficiaries). Positive associations between PM_2.5_ exposure and mortality from stroke, COPD, and T2DM were observed only among non-Medical Aid beneficiaries.

Sensitivity analyses examining associations using 24-month, 36-month, and 48-month moving averages of PM_2.5_ exposure are presented in Supplementary Material 11. HRs generally decreased with longer exposure windows, except for T2DM mortality. Associations for IHD, ALRI, and COPD mortality remained statistically significant after excluding deaths occurring within 3 months of baseline (Supplementary Material 12). For individuals with no residential moves, HRs were relatively higher, although trends remained similar overall (Supplementary Material 13). In two-pollutant models (adjusted for SO₂, NO₂, and O₃), HRs slightly increased for stroke, ALRI, and COPD mortality, while HR for IHD mortality decreased; however, statistical significance persisted across these models (Supplementary Material 14).

Table 4 reports excess deaths attributable to long-term PM_2.5_ exposure between 2010 and 2019. Due to the absence of significant associations, we did not estimate excess deaths from LC. The total number of excess deaths over the study period was estimated as follows: IHD, 13,696 (95% CI, 8,398 to 18,718); stroke, 8,765 (95% CI, 1,176 to 16,059); ALRI, 12,400 (95% CI, 6,562 to 17,969); COPD, 12,459 (95% CI, 8,390 to 16,231); and T2DM, 4,512 (95% CI, 741 to 8,009).

**Figure 2.**
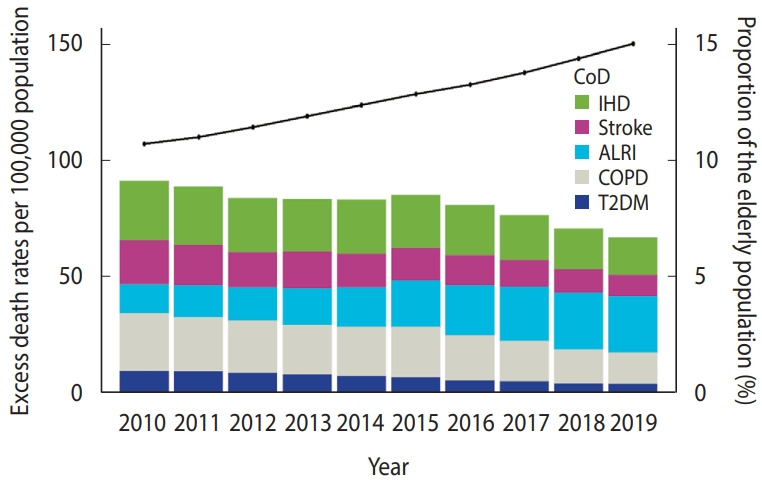
Rate of excess deaths attributed to the 12-month moving average particulate matter with an aerodynamic diameter <2.5 μm concentration in Korea per 100,000 people, and proportion of older adults in the population (%) from 2010 to 2019. CoD, cause of death; IHD, ischemic heart disease; ALRI, acute lower respiratory infection; COPD, chronic obstructive pulmonary disease; T2DM, type 2 diabetes mellitus.

Over the study period, excess mortality rates per 100,000 older adults due to long-term PM_2.5_ exposure increased for ALRI (12.6 in 2010 vs. 24.4 in 2019) but decreased for IHD, stroke, COPD, and T2DM (Figure 2, Supplementary Material 15). In 2019, mortality rates per 100,000 older adults attributable to PM_2.5_ exposure were 15.9 (95% CI, 9.8 to 21.8) for IHD, 9.3 (95% CI, 1.2 to 17.0) for stroke, 24.4 (95% CI, 12.9 to 35.3) for ALRI, 13.3 (95% CI, 8.9 to 17.4) for COPD, and 4.2 (95% CI, 0.7 to 7.5) for T2DM.

## DISCUSSION

### Long-term exposure to particulate matter with an aerodynamic diameter <2.5 μm and cause-specific mortalities

This study used a health-insurance-based retrospective cohort to follow the entire Korean population of older adults from 2010, evaluating associations between long-term PM_2.5_ exposure and 6 cause-specific mortalities. Our findings indicate that long-term PM_2.5_ exposure was associated with increased mortality from IHD, stroke, ALRI, COPD, and T2DM. The association between PM_2.5_ and IHD mortality was more pronounced in women than in men. Furthermore, associations of PM_2.5_ with COPD and T2DM were more clearly observed among the old-old population (≥75 years) compared to the young-old population (65-74 years).

We estimated the mortality burden attributable to long-term PM_2.5_ exposure. Due to population aging, the number of deaths attributable to long-term PM_2.5_ exposure increased from 4,888 in 2010 to 5,179 in 2019.

### Comparison with previous studies

This study estimated effect sizes between long-term PM_2.5_ exposure and cause-specific mortality, assuming linearity. We identified associations between long-term PM_2.5_ exposure and cause-specific mortality (IHD, stroke, ALRI, COPD, and T2DM), but not with LC. These findings are consistent with previous studies [1,3,4,11,12,15,28] (Supplementary Material 16); however, our estimated effect sizes were relatively lower compared to those in prior studies. We speculate this may be due to differences in regional characteristics, the study period, improvements in health conditions, and the implementation of PM_2.5_ reduction measures. Specifically, studies vary by countries or regions, population demographics (older adults vs. general population), and spatial scales (national, multinational, or specific metropolitan areas), influencing ambient PM_2.5_ concentration levels and distributions. For instance, Orellano et al. [15], adopted a global approach, encompassing various countries with diverse air pollution levels, whereas our study focused specifically on older adults across Korea. Differences in PM_2.5_ exposure levels, healthcare systems, policies, and underlying health conditions could therefore explain variations in effect sizes. Moreover, a previous study reported notable mortality increases associated with air pollution in 7 major Korean cities [3]. However, that study was restricted to urban areas, limiting its generalizability due to variations in PM_2.5_ levels, composition, and socioeconomic factors across regions.

### Gender differences

We found older women to be more susceptible to the adverse effects of long-term PM_2.5_ exposure, especially mortality from IHD, COPD, and T2DM. These results align with previous research [11,12,29]. Hormonal changes, such as the decline in estrogen levels after menopause, diminish estrogen’s cardiovascular protective effects, leading to impaired endothelial function, increased systemic inflammation, and heightened atherosclerosis risk [30]. Additionally, older women might have greater metabolic sensitivity, including increased oxidative stress and susceptibility to metabolic syndrome [31,32]. Differences in vascular structure, particularly the greater susceptibility of women to microvascular dysfunction, may further amplify the cardiovascular effects of PM_2.5_ exposure [33]. Exposure to air pollution promotes oxidative stress by increasing reactive oxygen species, which might lead to faster depletion of antioxidant defenses in women, exacerbating vascular and metabolic dysfunction [33].

Regarding COPD, non-infectious factors such as air pollution, smoking, and environmental exposures are primary triggers for disease onset [34]. Women typically have smaller lung capacities and narrower airways, resulting in higher particulate deposition relative to lung tissue volume, potentially intensifying the respiratory impact of air pollution [35]. Furthermore, lung function declines may progress more rapidly in women, increasing their susceptibility to COPD [36]. Post-menopausal hormonal changes might also influence systemic inflammation and respiratory health, further heightening COPD risk [37]. Psychosocial stress may also contribute, as older women generally report higher mental distress than older men [38], and stress is recognized as a potential risk factor for COPD [39].

### Age differences

Previous studies from the United States and Hong Kong reported stronger associations between long-term PM_2.5_ exposure and mortality from IHD and COPD in young-old adults [11,12]. In contrast, we found that older groups (≥75 years) were more susceptible to adverse PM_2.5_ effects, particularly for IHD, COPD, and T2DM mortality, compared to young-old adults (65-74 years). This discrepancy might be explained by several factors. Aging correlates with declines in lung and cardiovascular functions and reduced ability to cope with oxidative stress and inflammation, potentially increasing vulnerability to PM_2.5_’s harmful effects [40]. Variations in PM_2.5_ concentrations, lifestyles, regional differences, and ethnicity may also explain these discrepancies. For example, compared with the United States and Hong Kong, Korea experiences higher PM_2.5_ levels, potentially contributing to observed differences in susceptibility and health outcomes.

### Mortality burden due to long-term exposure to particulate matter with an aerodynamic diameter <2.5 μm

The estimated number of excess deaths and mortality rate from ALRI attributable to PM_2.5_ steadily increased from 2010 to 2019, with 2019 exhibiting the highest mortality burden among evaluated diseases (Figure 2). This trend likely reflects the rising proportion of older adults, given that respiratory diseases represent a leading cause of death in this demographic [41,42].

Between 2010 and 2015, the mortality rate from excess deaths due to PM_2.5_ exposure increased but declined from 2015 onward. The observed reduction from 2015 to 2019 is attributable to decreased mortality rates from IHD, stroke, COPD, and T2DM among individuals aged 65 and older. Advances in healthcare have improved disease management and reduced mortality, even in patients with these chronic conditions, thereby increasing life expectancy and decreasing death rates. However, ALRI-related mortality rates have increased, likely due to complications such as pneumonia in bedridden patients in long-term care facilities, where ALRI is frequently listed as the primary cause of death in Korea. Given the continuing population aging, the mortality burden from these 5 major diseases (IHD, stroke, ALRI, COPD, and T2DM) is unlikely to decrease substantially in the future. Consequently, efforts should focus on mitigating air pollution exposure. Preventive measures, including source control of particulate matter pollution and individual-level interventions (e.g., masks or air purifiers), could be critical for mitigating the long-term health impacts associated with PM_2.5_ exposure.

### Lung cancer mortality

Previous studies have reported significant associations between long-term PM_2.5_ exposure and LC mortality [3,4,15,28]. However, our study found a statistically significant but marginal negative association (HR, 0.972; 95% CI, 0.948 to 0.996), which could be attributed to several factors. First, detecting significant associations between long-term PM_2.5_ exposure and mortality may require extended observation periods. Since our study focused on associations using a 12-month moving average PM_2.5_ exposure, cumulative exposure might have been insufficiently captured. Additionally, our older study population may have included many advanced-stage LC cases, making it difficult to detect short-term effects of recent PM_2.5_ exposure.

### Sensitivity analyses

The results of our sensitivity analyses were generally consistent with those of the main analysis. When individuals who died within 1 month to 3 months of baseline were excluded, overall HRs decreased slightly, but statistically significant associations persisted for IHD, ALRI, and COPD (Supplementary Material 12). However, associations for stroke and T2DM lost statistical significance. This suggests the observed effects of long-term PM_2.5_ exposure remain robust even after excluding vulnerable populations. Conversely, failing to account for residential mobility slightly exaggerated effect sizes for all causes of death (Supplementary Material 13). This might be due to overestimating exposure levels when residential changes are ignored. Therefore, incorporating residential mobility is important for enhancing the accuracy of exposure assessments. Additionally, among older adults, shorter exposure windows (e.g., 1-year moving averages) were associated with higher HRs for IHD, stroke, ALRI, and COPD, but not LC or T2DM mortality (Supplementary Material 11). The reasons underlying this intriguing finding remain unclear but may involve factors such as underlying comorbidities or lifestyle changes. Future studies should explore these potential explanations more thoroughly. These results highlight the importance of considering residential mobility and additional demographic and environmental factors in future studies to comprehensively assess the health impacts of PM_2.5_ exposure.

### Strengths and limitations

To the best of our knowledge, this is the first study to evaluate associations between long-term PM_2.5_ exposure and cause-specific mortality in nearly the entire population of older adults in Korea. Comprehensive subgroup and sensitivity analyses enhance the robustness of our findings. Our results can be generalized to other countries with similar PM_2.5_ exposure levels and demographic characteristics. These findings provide valuable insights into the mortality burden among older adults attributable to long-term PM_2.5_ exposure, supporting policy formulation aimed at reducing PM_2.5_ levels and implementing appropriate interventions (e.g., wearing masks). Additionally, this study not only estimates HRs for cause-specific mortality but also quantifies excess deaths due to long-term PM_2.5_ exposure among older Koreans. Our estimates of excess mortality are substantially lower than global estimates provided by the Institute for Health Metrics and Evaluation using the Global Burden of Disease 2021 data. Specifically, excess death estimates from global studies are approximately 1.3 times to over 9.3 times higher than those reported in our study (Supplementary Material 17). This discrepancy suggests that global assessments, while useful for illustrating general disease burdens from air pollution, likely overestimate mortality due to inherent uncertainties. Therefore, our results offer a more precise estimation of the PM_2.5_-related mortality burden, informing targeted public health interventions and policies.

Nonetheless, this study has several limitations. First, we did not consider individual-level potential confounders, such as smoking, drinking, and physical activity status [43-45]. These factors could be potential confounders in the relationship between long-term exposure to PM_2.5_ and cause-specific mortality [46]. Additionally, residual confounding from unmeasured factors cannot be completely excluded. However, we minimized these issues by adjusting for district-level indicators as recommended by previous research [47]. Furthermore, the significantly lower participation rate of older Koreans in NHIS health screening compared to the general adult population limited our use of health screening data. We deliberately avoided incorporating such data to prevent selection bias, given potential differences between older participants and non-participants. Second, exposure measurement bias and misclassification may have occurred [48]. PM_2.5_ exposures in this study were assigned using the CMAQ model at a 9 km×9 km grid resolution, potentially failing to capture finer-scale individual exposure variations. This spatial limitation might introduce measurement error, particularly in environmentally heterogeneous areas. Future research should consider employing higher-resolution exposure data (e.g., 1×1 km grids) and integrating individual-level exposure metrics (e.g., time-varying patterns and agent-based modeling) to improve exposure assessment precision and better clarify associations between long-term air pollution exposure and health outcomes [49].

These measurement errors could attenuate observed associations, biasing results toward null findings and obscuring the true relationship between exposure and mortality outcomes.

Our findings elucidate the associations between long-term PM_2.5_ exposure and cause-specific mortality in Korea’s population of older adults from 2010 to 2019. We quantified the mortality burden attributable to long-term PM_2.5_ exposure among older Korean adults, providing crucial insights for targeted public health interventions.

## Figures and Tables

**Figure f3-epih-47-e2025028:**
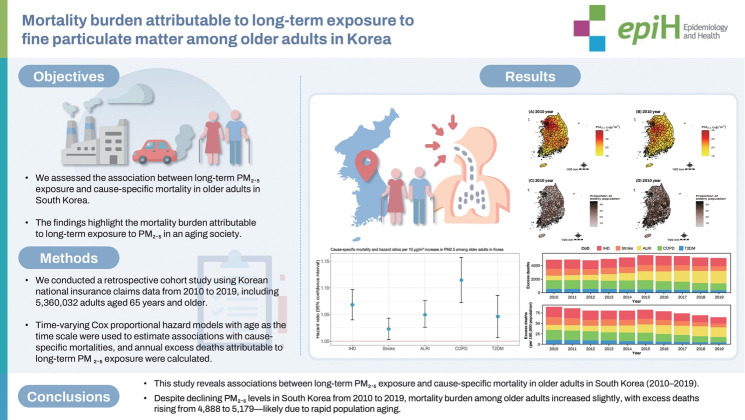


**Table 1.** Individual-level demographic characteristics of the study population in 2010

Characteristics	n (%)
Total	5,360,032 (100)
Age (yr)	
65-74	3,372,719 (62.9)
≥75	1,987,313 (37.1)
Gender	
Men	2,193,175 (40.9)
Women	3,166,857 (59.1)
Income level	
Medical Aid	466,464 (8.7)
Very low	709,070 (13.2)
Low	531,334 (9.9)
Mid	713,013 (13.3)
High	1,088,858 (20.3)
Very high	1,851,293 (34.5)
Cities and provinces	
Cities	
Seoul	981,866 (18.3)
Busan	396,438 (7.4)
Daegu	247,367 (4.6)
Incheon	234,427 (4.4)
Gwangju	126,303 (2.4)
Daejeon	126,494 (2.4)
Ulsan	76,950 (1.4)
Provinces	
Gyeonggi	993,858 (18.5)
Gangwon	211,265 (3.9)
Chungcheongbuk	198,059 (3.7)
Chungcheongnam	298,273 (5.6)
Jeollabuk	274,723 (5.1)
Jellanam	342,954 (6.4)
Gyeongsangbuk	408,935 (7.6)
Gyeongsangnam	377,975 (7.1)
Jeju	64,145 (1.2)
Underlying disease	
No	2,119,534 (39.5)
Yes	3,240,498 (60.5)

**Table 2.** Cause-specific deaths and mean survival time of the study population from 2010 to 2019

Cause of death	ICD-10	No. of deaths	Percentage of total deaths (n=1,774,563)	Percentage of the total study population (n=5,360,032)	Cause-specific mean event time, mean±SD (mo)
IHD	I20-I25	99,617	5.6	1.9	59.2±34.1
Stroke	I60-I69	180,930	10.2	3.4	56.8±34.3
ALRI	J12-J18, J20-J22	124,022	7.0	2.3	72.4±33.2
COPD	J40-J44	46,228	2.6	0.9	58.6±34.0
T2DM	E11	47,707	2.7	0.9	54.0±33.7
LC	C33-C34	110,520	6.2	2.3	59.2±34.4

ICD-10, International Classification of Diseases 10th revision; SD, standard deviation; IHD, ischemic heart disease; ALRI, acute lower respiratory infection; COPD, chronic obstructive pulmonary disease; LC, lung cancer; T2DM, type 2 diabetes mellitus.

**Table 3.** Cause-specific mortality associated with each 10 μg/m^3^ increase in the 12-month moving average PM_2.5_ concentrations among older adults^1^

Cause of death	Model 1	Model 2	Model 3	Model 4
IHD	1.238 (1.208, 1.268)	1.057 (1.030, 1.083)	1.053 (1.026, 1.079)	1.068 (1.040, 1.097)
Stroke	1.330 (1.307, 1.354)	1.062 (1.043, 1.082)	1.058 (1.038, 1.078)	1.023 (1.003, 1.043)
ALRI	0.861 (0.843, 0.880)	1.058 (1.034, 1.081)	1.054 (1.031, 1.078)	1.050 (1.026, 1.076)
COPD	1.261 (1.218, 1.306)	1.079 (1.042, 1.119)	1.075 (1.037, 1.114)	1.114 (1.072, 1.157)
LC	0.999 (0.976, 1.022)	0.950 (0.912, 0.990)	0.948 (0.926, 0.970)	0.972 (0.948, 0.996)
T2DM	1.490 (1.440, 1.542)	1.142 (1.102, 1.183)	1.134 (1.094, 1.175)	1.046 (1.007, 1.086)

Values are presented as hazard ratio (95% confidence interval). PM_2.5_, particulate matter with an aerodynamic diameter <2.5 μm; IHD, ischemic heart disease; ALRI, acute lower respiratory infection; COPD, chronic obstructive pulmonary disease; LC, lung cancer; T2DM, type 2 diabetes mellitus.

1 Model 1: unadjusted model; Model 2: adjusted for gender, age, type of insurance enrollment, income level, and strata (region); Model 3: adjusted for gender, age, type of insurance enrollment, income level, underlying disease, and strata (region); Model 4: adjusted for gender, age, type of insurance enrollment, income level, underlying disease, total population (district-level), proportion of older adults (≥65 years, district-level), education level (district-level), annual mean temperature (district-level), rainfall (district-level), smoking rate (district-level), and strata (region).

**Table 4.** Estimated number of excess deaths (95% confidence intervals) attributable to 12-month moving average PM_2.5_ concentrations among older adults in Korea, from 2010 to 2019^1^

Year	Total	Cause of death				
		IHD	Stroke	ALRI	COPD	T2DM
2010	4,888 (2,304, 7,323)	1,359 (835, 1,854)	1,009 (136, 1,847)	676 (359, 978)	1,315 (887, 1,709)	529 (87, 936)
2011	4,915 (2,329, 7,354)	1,381 (848, 1,885)	955 (128, 1,748)	766 (406, 1,109)	1,271(858, 1,654)	541 (89, 958)
2012	4,831 (2,318, 7,216)	1,329 (813, 1,819)	868 (116, 1,593)	825 (436, 1,197)	1,292 (868, 1,687)	516 (85, 919)
2013	5,035 (2,407, 7,518)	1,352 (830, 1,846)	950 (128, 1,739)	949 (503, 1,374)	1,283 (865, 1,670)	502 (82, 890)
2014	5,236 (2,548, 7,775)	1,446 (888, 1,975)	918 (123, 1,680)	1,074 (569, 1,554)	1,318 (889, 1,715)	481 (79, 852)
2015	5,586 (2,749, 8,267)	1,482 (909, 2,025)	911 (122, 1,669)	1,320 (699, 1,910)	1,393 (939, 1,813)	479 (79, 849)
2016	5,495 (2,726, 8,114)	1,468 (901, 2,005)	871 (117, 1,596)	1,459 (773, 2,113)	1,288 (868, 1,676)	408 (67, 724)
2017	5,426 (2,694, ,8,019)	1,370 (840, 1,874)	814 (109, 1,491)	1,647 (871, 2,387)	1,203 (810, 1,568)	393 (64, 698)
2018	5,241 (2,607, 7,752)	1,277 (781, 1,750)	751 (101, 1,379)	1,805 (953, 2,621)	1,069 (717, 1,398)	338 (55, 603)
2019	5,179 (2,585, 7,648)	1,230 (753, 1,685)	717 (96, 1,316)	1,880 (994, 2,727)	1,026 (689, 1,340)	325 (53, 580)
Total	51,832 (25,767, 76,986)	13,696 (8,398, 18,718)	8,765 (1,176, 16,059)	12,400 (6,562, 17,969)	12,459 (8,390, 16,231)	4,512 (741, 8,009)

PM_2.5_, particulate matter with an aerodynamic diameter <2.5 μm; IHD, ischemic heart disease; ALRI, acute lower respiratory infections; COPD, chronic obstructive pulmonary disease; T2DM, type 2 diabetes mellitus.

1 As long-term exposure to PM_2.5_ and lung cancer were not significantly associated, excess deaths from lung cancer due to long-term exposure to PM_2.5_ were not included in the calculation.

## Supplementary materials

Supplementary Material 1. Cause-specific deaths and ICD-10 codes are referenced in studies on associations between long-term exposure and cause-specific mortality

Supplementary Material 2. Scatter plots and correlation coefficients of monthly monitored PM_2.5_ and modeled PM_2.5_ (CMAQ) by city and province from 2015 to 2019

Supplementary Material 3. Description of exposure assessment

Supplementary Material 4. Comparison of Akaike Information Criterion across the different models

Supplementary Material 5. Flow diagram of the study population

Supplementary Material 6. Summary statistics of average (±SD) district-level indices from 2010 to 2019

Supplementary Material 7. Exposure–response curves for the association between the 12-month moving average exposure to PM_2.5_ and cause-specific mortality

Supplementary Material 8. HRs and 95% CIs for cause-specific mortality associated with a 10 μg/m^3^ increase in the 12-month moving average PM_2.5_ concentrations among elderly by gender

Supplementary Material 9. HRs and 95% CIs for cause-specific mortality associated with a 10 μg/m^3^ increase in the 12-month moving average PM_2.5_ concentrations among elderly according to age group

Supplementary Material 10. HRs and 95% CIs for cause-specific mortality associated with a 10 μg/m^3^ increase in the 12-month moving average PM_2.5_ concentrations among elderly by medical aid group

Supplementary Material 11. Associations between the 12-, 24-, 36, and 48-month moving average PM_2.5_ levels and cause-specific mortality among the elderly population

Supplementary Material 12. Association between long-term exposure to PM_2.5_ and cause-specific mortality, excluding individuals who died within 1, 2, and 3 months from baseline

Supplementary Material 13. Association between long-term exposure to PM_2.5_ and cause-specific mortality with unchanged residences across sixteen regions

Supplementary Material 14. Two-pollutant model results adjusted for SO_2_, NO_2_, and O_3_

Supplementary Material 15. Cause-specific excess death rates and 95% CIs per 100,000 population due to the 12-month moving average PM_2.5_ concentration in South Korea from 2010 to 2019

Supplementary Material 16. Summary of study designs and effect size associated with increased PM_2.5_ (per 10 μg/m^3^) on cause-specific mortality in the present study and related literature

Supplementary Material 17. Comparison of excess deaths due to PM_2.5_ between our study and the Institute for Health Metrics and Evaluation (IHME) using Global Burden of Disease 2021 data
