# Supplementary material for: Mortality burden attributable to long-term exposure to fine particulate matter among older adults in Korea
Source: Epidemiol Health. 2025 May 28;47:e2025028. doi: 10.4178/epih.e2025028 (PMC12425859; doi:10.4178/epih.e2025028)
Supplement: Supplementary Material 1. — Cause-specific deaths and ICD-10 codes are referenced in studies on associations between long-term exposure and cause-specific mortality [file epih-47-e2025028-Supplementary-1.docx]

Supplementary Material 1. Cause-specific deaths and ICD-10 codes are referenced in studies on associations between long-term exposure and cause-specific mortality.

| **Reference** | **IHD** | **Stroke** | **COPD** | **ALRI (including pneumonia)** | **LC** | **Diabetes** |
| --- | --- | --- | --- | --- | --- | --- |
| Burnett et al., 2014; | Yes | Yes | Yes | Yes | Yes | **-** |
| Burnett et al., 2018 | Yes | Yes | Yes | Yes | Yes | **-** |
| Chen and Hoek, 2020 | Yes (I20–I25) | Yes (I60–I69) | Yes (J40–J44, J47) | Yes (J12–J18, J20–J22) | Yes (C30–C39) | **-** |
| Lim et al., 2021 | Yes (I20–I25) | Yes (I60–I69) | Yes (J40–J44) |  | Yes (C30–C34) |  |
| Huang et al., 2019 | **-** | Yes (I60–I69) | **-** | **-** | **-** | **-** |
| Kim et al., 2020 | **-** | **-** | **-** | Yes (J12–J18, J20–J22) |  |  |
| Thacher et al., 2021 | **-** | **-** | **-** | **-** | **-** | Yes (E11) |
| Turner et al., 2011 | Yes (I20–I25) | Yes (I60–I69) | Yes(J19–J46*) | Yes (J12–J18) |  | Yes (E10–E14) |
| Wang et al., 2020 | Yes | Yes | Yes | Yes | Yes |  |
| Wong et al., 2015 | Yes (I20–I25) | Yes (I60–I69) | J40–J44, J47 | Yes (J12–J18) | **-** | **-** |

***** COPD and allied conditions were also considered.

**Abbreviations:** ICD-10, International Classification of Diseases; IHD, ischemic heart disease; ALRI, acute lower respiratory infection; COPD, chronic obstructive pulmonary disease; LC, lung cancer.
