# Supplementary material for: Mortality burden attributable to long-term exposure to fine particulate matter among older adults in Korea
Source: Epidemiol Health. 2025 May 28;47:e2025028. doi: 10.4178/epih.e2025028 (PMC12425859; doi:10.4178/epih.e2025028)
Supplement: Supplementary Material 2. — Scatter plots and correlation coefficients of monthly monitored PM2.5 and modeled PM2.5 (CMAQ) by city and province from 2015 to 2019 [file epih-47-e2025028-Supplementary-2.docx]

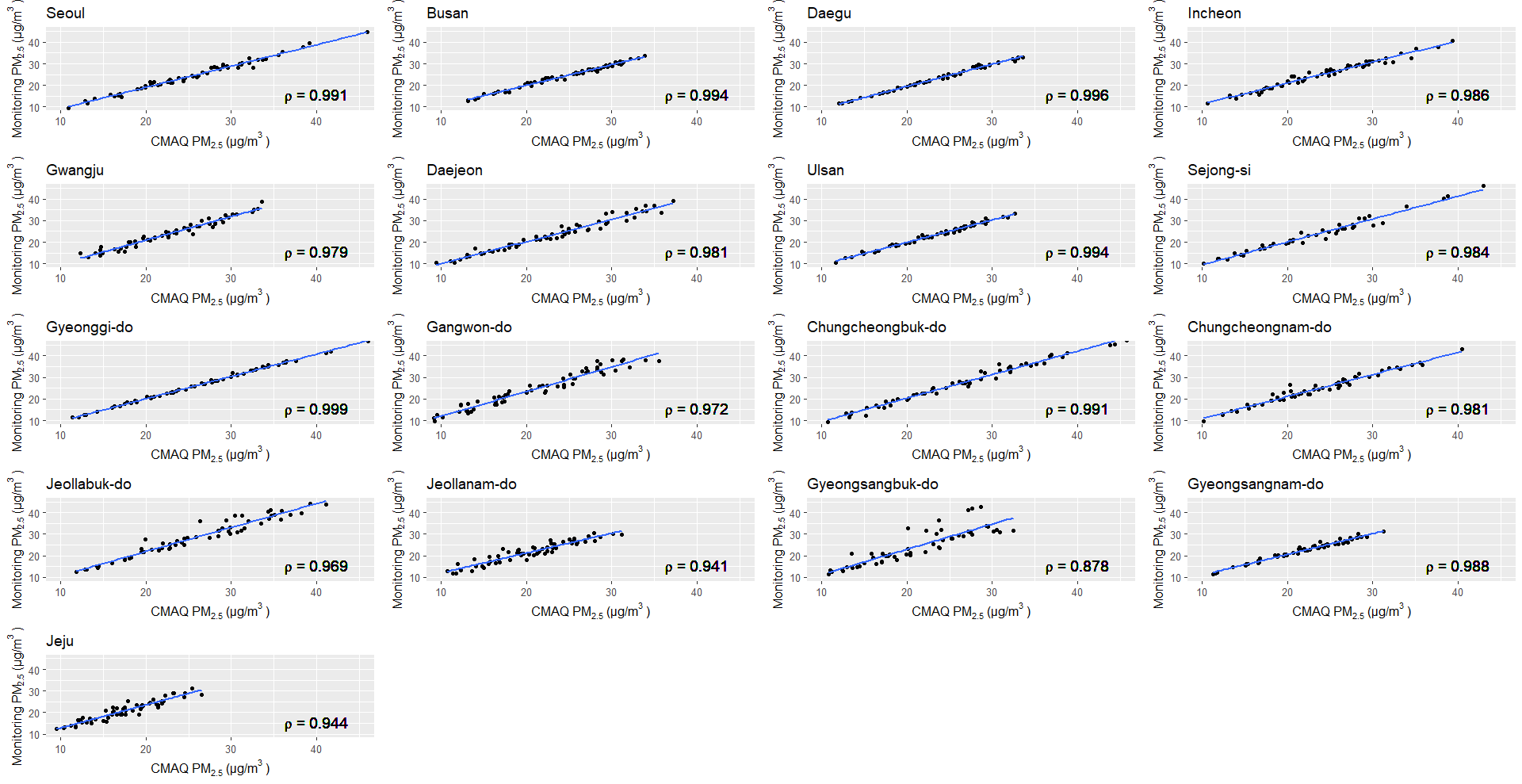


Supplementary Material 2. Scatter plots and correlation coefficients of monthly monitored PM_2.5_ and modeled PM_2.5_ (CMAQ) by city and province from 2015 to 2019.
