# Supplementary material for: Mortality burden attributable to long-term exposure to fine particulate matter among older adults in Korea
Source: Epidemiol Health. 2025 May 28;47:e2025028. doi: 10.4178/epih.e2025028 (PMC12425859; doi:10.4178/epih.e2025028)
Supplement: Supplementary Material 3. — Description of exposure assessment [file epih-47-e2025028-Supplementary-3.docx]

Supplementary Material 3. Description of exposure assessment

Due to the absence of nationwide PM_2.5_ monitoring in South Korea before 2015, this study used modeled PM_2.5_ data. The advantage of modeling data is that, unlike measurement data, it can generate data for all spatiotemporal points (i.e., administrative districts) without missing values. We utilized the Community Multiscale Air Quality (CMAQ, version 4.7.1) model recommended by the U.S. Environmental Protection Agency (https://www.epa.gov/cmaq). The CMAQ models were incorporated with domestic emissions data from the Clean Air Policy Support System 2010 and 2016 and foreign emissions data from the Model Inter-Comparison Study 2010, CREATE 2015, [1] and KORUSv5. [2] Meteorological inputs were prepared using the Weather Research and Forecasting System (version 3.4.1), [3] with initial fields obtained from the Final Operational Global Analysis Data reanalysis by the National Oceanic and Atmospheric Administration. [4] The meteorological chemistry interface processor (version 3.6) was used to prepare the meteorological inputs for the CMAQ emissions. Anthropogenic data from the Korean National Emissions Inventory and biogenic data from the Model of Emissions of Gases and Aerosols from Nature were processed using the Sparse Matrix Operator Kernal Emissions. [5] Two modeling domains with horizontal resolutions of 27 and 9 km were employed. The 9-km grid covered South Korea (67$\times$82 cells), and the 27-km grid encompassed Northeast Asia to account for regional impacts. The boundary conditions for the 9-km domain were derived from the simulation of the coarse 27-km domain. [6] The gridded hourly PM_2.5_ concentrations from the CMAQ simulations were resampled for each administrative area in South Korea using Geographic Information System shape files

1.Woo J-H, Kim Y, Kim H-K, Choi K-C, Eum J-H, Lee J-B, et al. Development of the CREATE Inventory in Support of Integrated Climate and Air Quality Modeling for Asia. Sustainability 2020;12

2.Woo J, Kim Y, Kim J, Park M, Jang Y, Kim J, et al. KORUS Emissions: A comprehensive Asian emissions information in support of the NASA/NIER KORUS-AQ mission. Elementa: Science of the Anthropocene 2021

3.Skamarock WC, Klemp JB, Dudhia J, Gill DO, Barker DM, Duda MG, et al. A description of the advanced research WRF version 3. NCAR technical note 2008;475:10.5065.

4.Li Q, Wei M, Wang Z, Chu Y, Ma L. Evaluation and correction of ground-based microwave radiometer observations based on NCEP-FNL data. Atmospheric and Climate Sciences 2019;9:229-242.

5.Benjey W, Houyoux M, Susick J. Implementation of the SMOKE emission data processor and SMOKE tool input data processor in models-3. US EPA 2001

6.Bae M, Kim B-U, Kim HC, Woo JH, Kim S. An observation-based adjustment method of regional contribution estimation from upwind emissions to downwind PM2.5 concentrations. Environment International 2022;163
