# Supplementary material for: Mortality burden attributable to long-term exposure to fine particulate matter among older adults in Korea
Source: Epidemiol Health. 2025 May 28;47:e2025028. doi: 10.4178/epih.e2025028 (PMC12425859; doi:10.4178/epih.e2025028)
Supplement: Supplementary Material 4. — Comparison of Akaike Information Criterion across the different models [file epih-47-e2025028-Supplementary-4.docx]

Supplementary Material 4. Comparison of Akaike Information Criterion across the different models.

| **Model** | **IHD** | **Stroke** | **ALRI** | **COPD** | **LC** | **T2DM** |
| --- | --- | --- | --- | --- | --- | --- |
| Model 1 | 2258800·9 | 4132302·6 | 2727958·7 | 1038561·1 | 2607762·7 | 1111159·1 |
| Model 2 | 2249663·8 | 4111688·6 | 2701534·5 | 1008465·0 | 2550036·2 | 1111671·5 |
| Model 3 | 2243549·7 | 4097449·8 | 2697337·5 | 1006193·7 | 2540234·9 | 1059914·3 |
| Model 4 | 2243308·0 | 4096681·2 | 2697212·6 | 1005964·5 | 2539919·6 | 1059492·2 |

**Abbreviations:** IHD, ischemic heart disease; ALRI, acute lower respiratory infections; COPD, chronic obstructive pulmonary disease; LC, lung cancer; T2DM, type 2 diabetes mellitus.

Model 1: unadjusted model

Model 2: adjusted for gender, age, type of insurance enrollment, income level, and strata (region)

Model 3: adjusted for gender, age, type of insurance enrollment, income level, underlying disease, and strata (region)

Model 4: adjusted for gender, age, type of insurance enrollment, income level, underlying disease, number of population, proportion of elderly (≥65), education level, temperature, rainfall, smoking rate, and strata (region)
