# Supplementary figures and images for: Mortality burden attributable to long-term exposure to fine particulate matter among older adults in Korea
Source: Epidemiol Health. 2025 May 28;47:e2025028. doi: 10.4178/epih.e2025028 (PMC12425859; doi:10.4178/epih.e2025028)

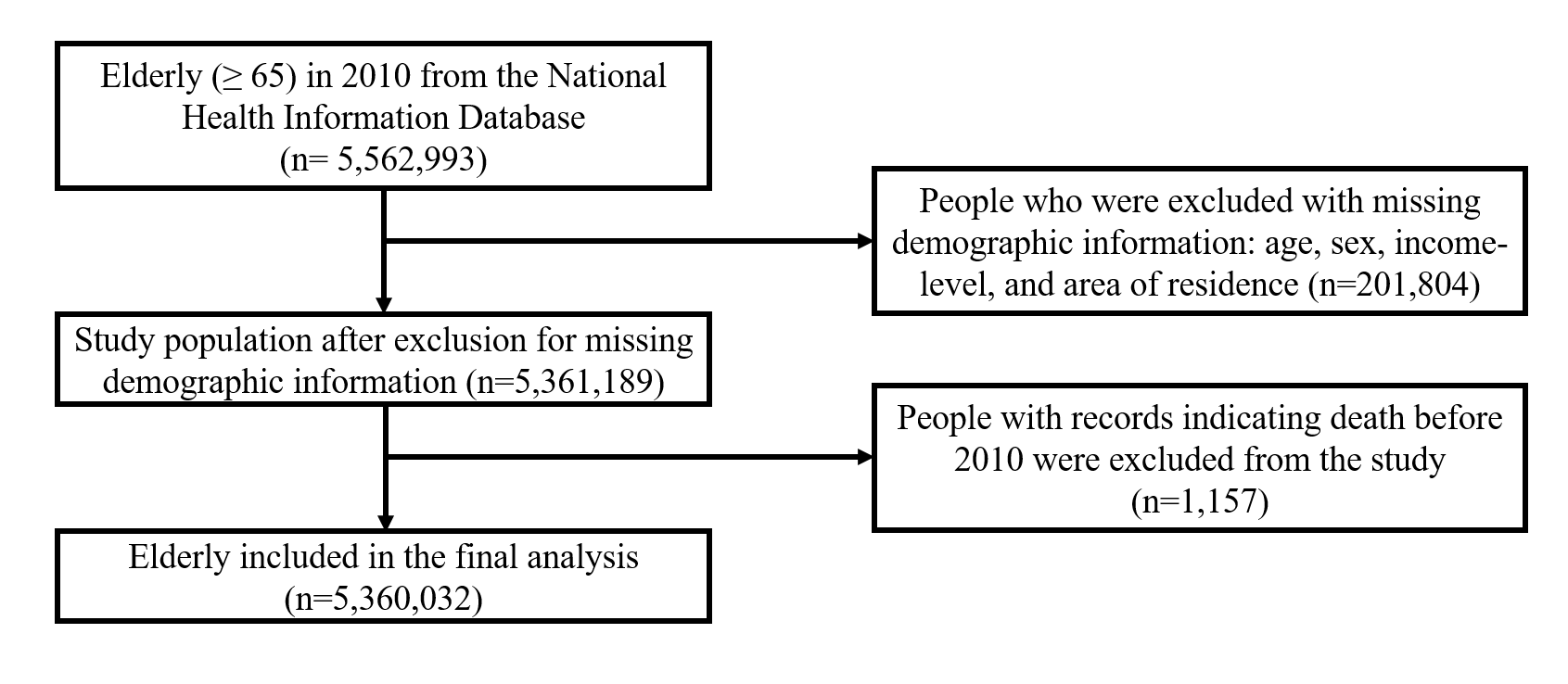


Supplementary Material 5. Flow diagram of the study population

Supplement: Supplementary Material 5. — Flow diagram of the study population [file epih-47-e2025028-Supplementary-5.docx]
