# Supplementary material for: Mortality burden attributable to long-term exposure to fine particulate matter among older adults in Korea
Source: Epidemiol Health. 2025 May 28;47:e2025028. doi: 10.4178/epih.e2025028 (PMC12425859; doi:10.4178/epih.e2025028)
Supplement: Supplementary Material 6. — Summary statistics of average (±SD) district-level indices from 2010 to 2019 [file epih-47-e2025028-Supplementary-6.docx]

Supplementary Material 6. Summary statistics of average ($\pm$SD) district-level indices from 2010 to 2019.

| **Year** | **Number of population** | **Proportion of elderly (%)** | **Proportion of high school diplomas (%)** | **Smoking rates (%)** | **Average temperature (°C)** | **Average rainfall (mm)** |
| --- | --- | --- | --- | --- | --- | --- |
| 2010 | 202062·7 (159330·5) | 15·2 (7·4) | 31·7 (13·7) | 25·3 (2·6) | 12·3 (1·4) | 1481·0 (284·3) |
| 2011 | 202937·1 (159852·0) | 15·5 (7·4) | - | 24·8 (2·7) | 11·9 (1·4) | 1622 (309·4) |
| 2012 | 203793·1 (160516·6) | 16·1 (7·5) | - | 24·6 (2·6) | 11·9 (1·4) | 1525·6 (267·2) |
| 2013 | 204565·8 (161146·6) | 16·6 (7·6) | - | 24·1 (2·6) | 12·3 (1·5) | 1185·7 (230·9) |
| 2014 | 205311·7 (161724·4) | 17·1 (7·7) | - | 23·8 (2·7) | 12·6 (1·3) | 1110·1 (363·1) |
| 2015 | 206117·4 (162298·8) | 17·6 (7·8) | 37·0 (13·2) | 22·2 (2·7) | 13·0 (1·2) | 947·7 (291·5) |
| 2016 | 206784·9 (162782·7) | 18·0 (7·8) | - | 22·3 (2·7) | 13·2 (1·3) | 1232·7 (351·6) |
| 2017 | 207114·2 (163390·3) | 18·8 (7·9) | - | 21·6 (2·7) | 12·6 (1·4) | 971·0 (196·6) |
| 2018 | 207304·2 (164659·3) | 19·4 (8·0) | - | 21·6 (2·8) | 12·5 (1·4) | 1394·2 (235·2) |
| 2019 | 207399·4 (166028·8) | 20·3 (8·2) |  | 20·3 (3·1) | 13·0 (1·3) | 1149·1 (346·8) |
| Total | 205339·0 (161903·5) | 17·5 (7·9) | 37·1 (14·0) | 23·1 (3·1) | 12·5 (1·4) | 1262·0 (366·6) |
