# Supplementary material for: Mortality burden attributable to long-term exposure to fine particulate matter among older adults in Korea
Source: Epidemiol Health. 2025 May 28;47:e2025028. doi: 10.4178/epih.e2025028 (PMC12425859; doi:10.4178/epih.e2025028)
Supplement: Supplementary Material 7. — Exposure–response curves for the association between the 12-month moving average exposure to PM2.5 and cause-specific mortality [file epih-47-e2025028-Supplementary-7.docx]

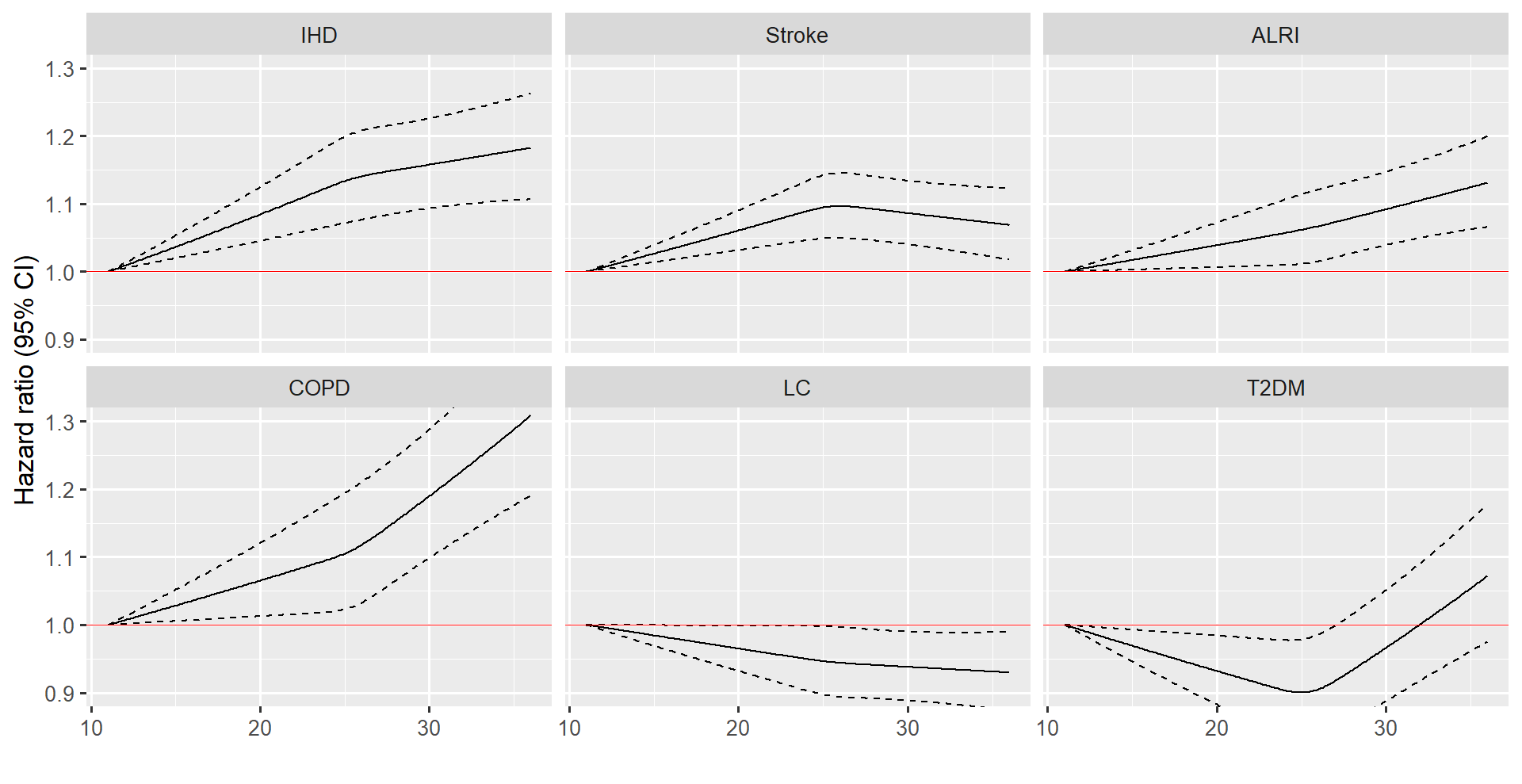


Supplementary Material 7**.** Exposure–response curves for the association between the 12-month moving average exposure to PM_2.5_ and cause-specific mortality

**Abbreviations**: CI, confidence interval; IHD, ischemic heart disease; ALRI, acute lower respiratory infection; COPD, chronic obstructive pulmonary disease; LC, lung cancer; T2DM, type 2 diabetes mellitus.
