# Supplementary material for: Mortality burden attributable to long-term exposure to fine particulate matter among older adults in Korea
Source: Epidemiol Health. 2025 May 28;47:e2025028. doi: 10.4178/epih.e2025028 (PMC12425859; doi:10.4178/epih.e2025028)
Supplement: Supplementary Material 8. — HRs and 95% CIs for cause-specific mortality associated with a 10 μg/m3 increase in the 12-month moving average PM2.5 concentrations among elderly by gender [file epih-47-e2025028-Supplementary-8.docx]

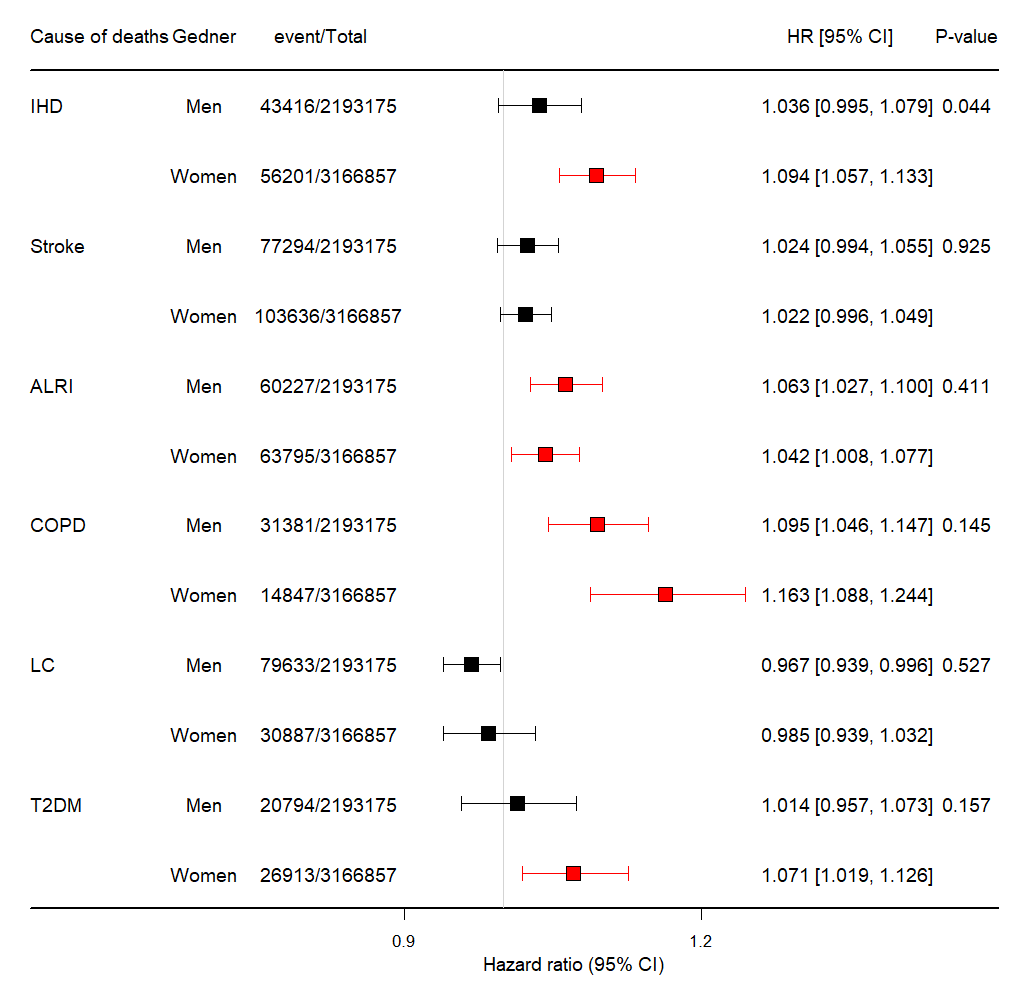


Supplementary Material 8**.** HRs and 95% CIs for cause-specific mortality associated with a 10 μg/m^3^ increase in the 12-month moving average PM_2.5_ concentrations among elderly by gender. All models were adjusted for gender, age, type of insurance enrollment, income level, underlying disease, number of population, proportion of elderly (≥65), education level, temperature, rainfall, smoking rate, and strata (region). A t-test was conducted to assess the difference in effect size between the two groups in the subgroup analyses.

**Abbreviations:** HR, hazard ratio; CI, confidence interval; IHD, ischemic heart disease; ALRI, acute lower respiratory infection; COPD, chronic obstructive pulmonary disease; LC, lung cancer; T2DM, type 2 diabetes mellitus.
