# Supplementary material for: Mortality burden attributable to long-term exposure to fine particulate matter among older adults in Korea
Source: Epidemiol Health. 2025 May 28;47:e2025028. doi: 10.4178/epih.e2025028 (PMC12425859; doi:10.4178/epih.e2025028)
Supplement: Supplementary Material 11. — Associations between the 12-, 24-, 36, and 48-month moving average PM2.5 levels and cause-specific mortality among the elderly population [file epih-47-e2025028-Supplementary-11.docx]

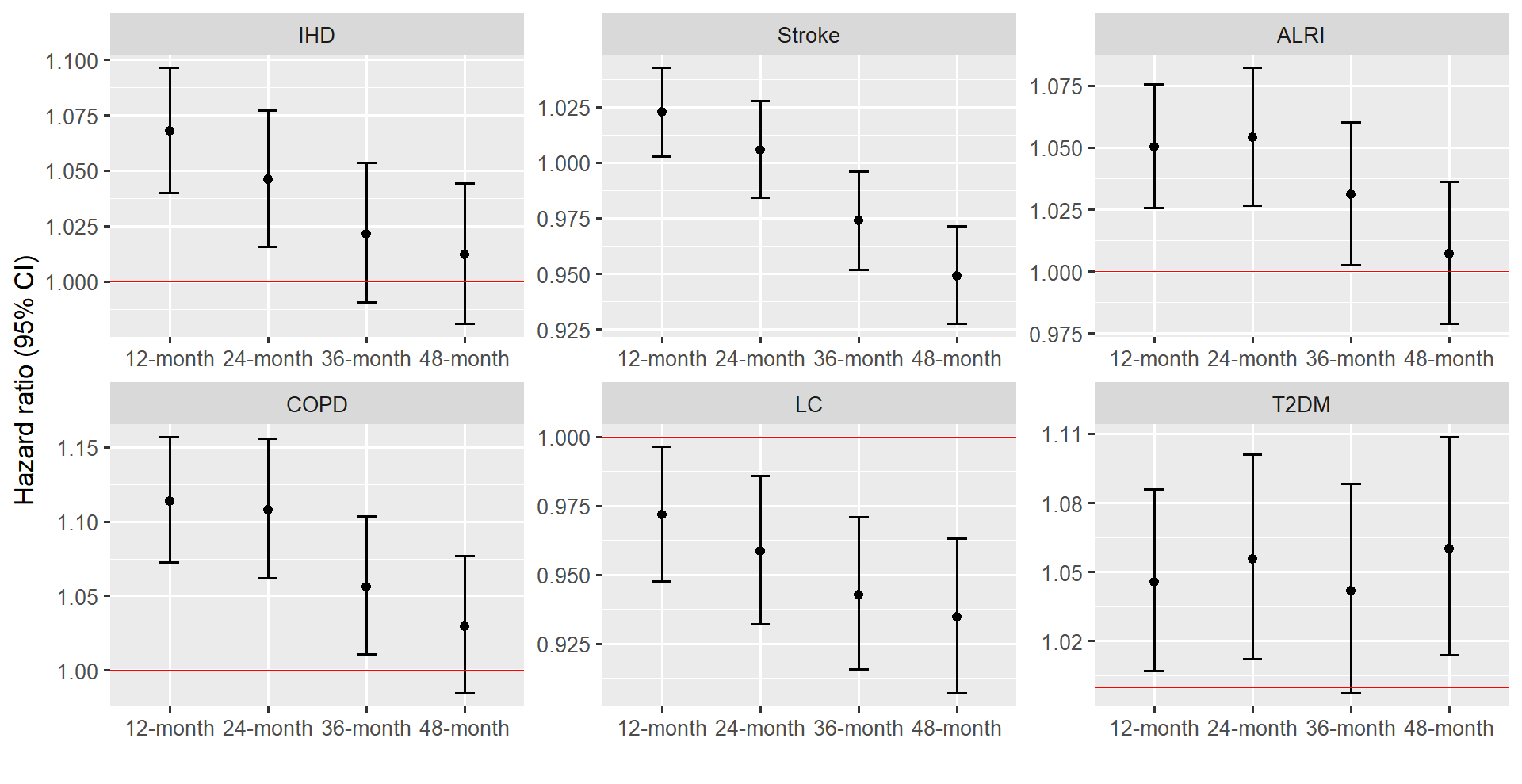


Supplementary Material 11**.** Associations between the 12-, 24-, 36, and 48-month moving average PM_2.5_ levels and cause-specific mortality among the elderly population.

**Abbreviations:** CI, confidence interval; IHD, ischemic heart disease; ALRI, acute lower respiratory infections; COPD, chronic obstructive pulmonary disease; LC, lung cancer; T2DM, type 2 diabetes mellitus.
