# Supplementary material for: Mortality burden attributable to long-term exposure to fine particulate matter among older adults in Korea
Source: Epidemiol Health. 2025 May 28;47:e2025028. doi: 10.4178/epih.e2025028 (PMC12425859; doi:10.4178/epih.e2025028)
Supplement: Supplementary Material 12. — Association between long-term exposure to PM2.5 and cause-specific mortality, excluding individuals who died within 1, 2, and 3 months from baseline [file epih-47-e2025028-Supplementary-12.docx]

Supplementary Material 12**.** Association between long-term exposure to PM_2.5_ and cause-specific mortality, excluding individuals who died within 1, 2, and 3 months from baseline.

| **Cause of death** | **Main model** | | **1-month** | | **2-month** | | **3-month** | |
| --- | --- | --- | --- | --- | --- | --- | --- | --- |
|  | **HR** | **95% CI** | **HR** | **95% CI** | **HR** | **95% CI** | **HR** | **95% CI** |
| IHD | **1.068** | **1.040, 1.097** | **1.057** | **1.030, 1.086** | **1.051** | **1.024, 1.080** | **1.040** | **1.012, 1.068** |
| Stroke | **1.023** | **1.003, 1.043** | 1.013 | 0.993, 1.033 | 1.004 | 0.984, 1.024 | 0.993 | 0.973, 1.013 |
| ALRI | **1.050** | **1.026, 1.076** | **1.047** | **1.022, 1.072** | **1.043** | **1.018, 1.068** | **1.038** | **1.014, 1.063** |
| COPD | **1.114** | **1.072, 1.157** | **1.105** | **1.064, 1.148** | **1.099** | **1.058, 1.142** | **1.089** | **1.048, 1.132** |
| LC | 0.972 | 0.948, 0.996 | 0.965 | 0.941, 0.990 | 0.956 | 0.932, 0.981 | 0.948 | 0.924, 0.972 |
| T2DM | **1.046** | **1.007, 1.086** | 1.038 | 0.999, 1.078 | 1.024 | 0.985, 1.063 | 1.012 | 0.974, 1.052 |

**Abbreviations:** HR, hazard ratio; CI, confidence interval; IHD, ischemic heart disease; ALRI, acute lower respiratory infection; COPD, chronic obstructive pulmonary disease; LC, lung cancer; T2DM, type 2 diabetes mellitus.
