# Supplementary material for: Mortality burden attributable to long-term exposure to fine particulate matter among older adults in Korea
Source: Epidemiol Health. 2025 May 28;47:e2025028. doi: 10.4178/epih.e2025028 (PMC12425859; doi:10.4178/epih.e2025028)
Supplement: Supplementary Material 13. — Association between long-term exposure to PM2.5 and cause-specific mortality with unchanged residences across sixteen regions [file epih-47-e2025028-Supplementary-13.docx]

Supplementary Material 13**.** Association between long-term exposure to PM_2.5_ and cause-specific mortality with unchanged residences across sixteen regions.

| **Cause of death** | **Main model** | | **Unchanged residences** | |
| --- | --- | --- | --- | --- |
|  | **HR** | **95% CI** | **HR** | **95% CI** |
| IHD | **1.068** | **1.040, 1.097** | **1.083** | **1.053, 1.114** |
| Stroke | **1.023** | **1.003, 1.043** | **1.062** | **1.040, 1.084** |
| ALRI | **1.050** | **1.026, 1.076** | **1.080** | **1.053, 1.108** |
| COPD | **1.114** | **1.072, 1.157** | **1.145** | **1.100, 1.191** |
| LC | 0.972 | 0.948, 0.996 | 1.008 | 0.982, 1.034 |
| T2DM | **1.046** | **1.007, 1.086** | **1.064** | **1.022, 1.107** |

**Abbreviations:** HR, hazard ratio; CI, confidence interval; IHD, ischemic heart disease; ALRI, acute lower respiratory infection; COPD, chronic obstructive pulmonary disease; LC, lung cancer; T2DM, type 2 diabetes mellitus.
