# Supplementary material for: Mortality burden attributable to long-term exposure to fine particulate matter among older adults in Korea
Source: Epidemiol Health. 2025 May 28;47:e2025028. doi: 10.4178/epih.e2025028 (PMC12425859; doi:10.4178/epih.e2025028)
Supplement: Supplementary Material 14. — Two-pollutant model results adjusted for SO2, NO2, and O3 [file epih-47-e2025028-Supplementary-14.docx]

Supplementary Material 14**.** Two-pollutant model results adjusted for SO_2_, NO_2_, and O_3_.

| **Cause of death** | **Main model** | | **Adjusted for SO_2_** | | **Adjusted for NO_2_** | | **Adjusted for O_3_** | |
| --- | --- | --- | --- | --- | --- | --- | --- | --- |
|  | **HR** | **95% CI** | **HR** | **95% CI** | **HR** | **95% CI** | **HR** | **95% CI** |
| IHD | **1.068** | **1.040, 1.097** | **1.046** | **1.018, 1.075** | **1.057** | **1.029, 1.086** | **1.064** | **1.036, 1.094** |
| Stroke | **1.023** | **1.003, 1.043** | **1.024** | **1.004, 1.045** | **1.033** | **1.013, 1.053** | **1.038** | **1.017, 1.059** |
| ALRI | **1.050** | **1.026, 1.076** | **1.074** | **1.048, 1.100** | **1.054** | **1.030, 1.079** | **1.059** | **1.034, 1.085** |
| COPD | **1.114** | **1.072, 1.157** | **1.118** | **1.076, 1.162** | **1.163** | **1.119, 1.209** | **1.162** | **1.117, 1.209** |
| LC | 0.972 | 0.948, 0.996 | 0.975 | 0.950, 1.100 | 0.989 | 0.965, 1.015 | 0.989 | 0.964, 1.015 |
| T2DM | **1.046** | **1.007, 1.086** | **1.049** | **1.010, 1.091** | **1.042** | **1.003, 1.082** | **1.059** | **1.019, 1.101** |

**Abbreviations:** HR, hazard ratio; CI, confidence interval; SO_2_, sulfur dioxide; NO_2_, nitrogen dioxide; O_3_, ozone; IHD, ischemic heart disease; ALRI, acute lower respiratory infection; COPD, chronic obstructive pulmonary disease; LC, lung cancer; T2DM, type 2 diabetes mellitus.
