# Supplementary material for: Mortality burden attributable to long-term exposure to fine particulate matter among older adults in Korea
Source: Epidemiol Health. 2025 May 28;47:e2025028. doi: 10.4178/epih.e2025028 (PMC12425859; doi:10.4178/epih.e2025028)
Supplement: Supplementary Material 15. — Cause-specific excess death rates and 95% CIs per 100,000 population due to the 12-month moving average PM2.5 concentration in South Korea from 2010 to 2019 [file epih-47-e2025028-Supplementary-15.docx]

Supplementary Material 15**.** Cause-specific excess death rates and 95% CIs per 100,000 population due to the 12-month moving average PM_2.5_ concentration in South Korea from 2010 to 2019.

| Year | Excess deaths per 100,000 population (95% CIs) | | | | |
| --- | --- | --- | --- | --- | --- |
|  | IHD | Stroke | ALRI | COPD | T2DM |
| 2010 | 25.4 (15.6–34.7) | 18.9 (2.5–34.5) | 12.6 (6.7–18.3) | 24.6 (16.6–31.9) | 9.9 (1.6–17.5) |
| 2011 | 25.0 (15.3–34.1) | 17.3 (2.3–31.6) | 13.9 (7.4–20.1) | 23.0 (15.5–29.9) | 9.8 (1.6–17.3) |
| 2012 | 23.1 (14.1­–31.6) | 15.1 (2.0–27.7) | 14.3 (7.6–20.8) | 22.4 (15.1–29.3) | 9.0 (1.5–16.0) |
| 2013 | 22.4 (13.8–30.6) | 15.7 (2.1–28.8) | 15.7 (8.3–22.8) | 21.3 (14.3–27.7) | 8.3 (1.4–14.8 |
| 2014 | 23.0 (14.1–31.4) | 14.6 (2.0–26.7) | 17.0 (9.0–24.7) | 20.9 (14.1–27.2) | 7.6 (1.3–13.5) |
| 2015 | 22.6 (13.9–30.9) | 13.9 (1.9–25.5) | 20.1 (10.7–29.2) | 21.3 (14.3–27.7) | 7.3 (1.2–13.0) |
| 2016 | 21.7 (13.3–29.6) | 12.9 (1.7–23.5) | 21.5 (11.4–31.2) | 19.0 (12.8–24.7) | 6.0 (1.0–10.7) |
| 2017 | 19.4 (11.9–26.5) | 11.5 (1.5–21.1) | 23.3 (12.3–33.8) | 17.0 (11.5–22.2) | 5.6 (0.9–9.9) |
| 2018 | 17.3 (10.6–23.7) | 10.2 (1.4–18.7) | 24.4 (12.9–35.5) | 14.5 (9.7–18.9) | 4.6 (0.7–8.2) |
| 2019 | 15.9 (9.8–21.8) | 9.3 (1.2–17.0) | 24.4 (12.9–35.3) | 13.3 (8.9–17.4) | 4.2 (0.7–7.5) |

**Abbreviations:** CI, confidence interval; IHD, ischemic heart disease; ALRI, acute lower respiratory infections; COPD, chronic obstructive pulmonary disease; LC, lung cancer; T2DM, type 2 diabetes mellitus.

**Note:** As long-term exposure to PM_2.5_ and lung cancer mortality have no statistically significant positive association, excess deaths for lung cancer due to long-term exposure to PM_2.5_ were not included in the calculation.
