# Supplementary material for: Mortality burden attributable to long-term exposure to fine particulate matter among older adults in Korea
Source: Epidemiol Health. 2025 May 28;47:e2025028. doi: 10.4178/epih.e2025028 (PMC12425859; doi:10.4178/epih.e2025028)
Supplement: Supplementary Material 16. — Summary of study designs and effect size associated with increased PM2.5 (per 10 μg/m3) on cause-specific mortality in the present study and related literature [file epih-47-e2025028-Supplementary-16.docx]

| **Study** | ***This study*** | ***Orellano et al., 2024*** | ***Chen and Hoek, 2020*** | ***Lim et al., 2020*** | ***Strak et al., 2021*** | ***Turner et al., 2017*** | ***Wang et al., 2020*** | ***Wong et al., 2015*** |
| --- | --- | --- | --- | --- | --- | --- | --- | --- |
| Study design | Cohort | Systematic review and meta-analysis | Systematic review and meta-analysis | Cohort | Eight Cohorts (pooled analysis) | Cohort | Cohort | Cohort |
| Study period | 2010–2019 | Up to May 2023 | Up to 9 October 2018 | 2009–2016 | 1989–2015 | 1998–2004 | 2000–2008 | 1999–2011 |
| Number of study subjects | 5,360,032 | 106 | 107 | 1,720,230 | 325,367 | 669,046 | 53 million | 59,362 |
| Population | Elderly (65+) | General population | General population | Elderly (65+) | General population | 40+ | Elderly (65+) | Elderly (65+) |
| Country | South Korea |  | - | South Korea | Europe | U.S | U.S. | Hong Kong |
| Region | Nationwide | Global | Global | Seven metropolitan cities | Multi-country | Nationwide | Nationwide | 18 districts |
| ***Cause of deaths*** | **Hazard ratio (95% CI)** | | | | | | | |
| IHD | 1·07 (1·04, 1·10) | 1·14 (1·10, 1·19) | 1·16 (1·10, 1·21) | 1·31 (1·23, 1·38) | 1·11 (1·06, 1·17) | 1·06 (1·02, 1·09) | 1·13 (1·11, 1·14) | 1·42 (1·16, 1·73) |
| Stroke | 1·02 (1·00, 1·04) | 1·15 (1·10, 1·19) | 1·11 (1·04, 1·18) | 1·02 (0·97, 1·06) | 1·13 (1·05, 1·21) | 1·13 (1·06, 1·21) | 1·13 (1·10, 1·15) | 1·24 (1·00, 1·53) |
| ALRI | 1·05 (1·03, 1·08) | 1·20 (1·10, 1·33) | 1·16 (1·01, 1·34) | - | - | 1·24 (1·12, 1·37) | 1·08 (1·04, 1·11) | 0·94 (0·77, 1·14) |
| COPD | 1·11 (1·07, 1·16) | 1·14 (1·08, 1·20) | 1·11 (1·05, 1·17) | 1·10 (1·00, 1·20) | 1·13 (1·00, 1·28) | 1·06 (0·97, 1·15) | 1·02 (0·99, 1·05) | 1·30 (0·98, 1·74) |
| LC | 0·97 (0·95, 1·00) | 1·09 (1·05, 1·14) | 1·12 (1·07, 1·16) | 1·07 (1·01, 1·13) | - | 1·13 (1·06, 1·21) | 1·00 (0·97, 1·02) | - |
| T2DM | 1·05 (1·01, 1·09) | - | - | - | 1·15 (1·11, 1·19)^a)^ | 1·01 (0·90, 1·15) ^a)^ | - | - |

Supplementary Material 16**.** Summary of study designs and effect size associated with increased PM_2.5_ (per 10 μg/m^3^) on cause-specific mortality in the present study and related literature.

^a)^ In these studies, diabetes included both type 1 (E10) and type 2 (E11-E14).

**Abbreviations:** CI, confidence interval; IHD, ischemic heart disease; ALRI, acute lower respiratory infections; COPD, chronic obstructive pulmonary disease; LC, lung cancer; T2DM, type 2 diabetes mellitus
