# Supplementary material for: Mortality burden attributable to long-term exposure to fine particulate matter among older adults in Korea
Source: Epidemiol Health. 2025 May 28;47:e2025028. doi: 10.4178/epih.e2025028 (PMC12425859; doi:10.4178/epih.e2025028)
Supplement: Supplementary Material 17. — Comparison of excess deaths due to PM2.5 between our study and the Institute for Health Metrics and Evaluation (IHME) using Global Burden of Disease 2021 data [file epih-47-e2025028-Supplementary-17.docx]

Supplementary Material 17**.** Comparison of excess deaths due to PM_2.5_ between our study and the Institute for Health Metrics and Evaluation (IHME) using Global Burden of Disease 2021 data

| Year | Excess deaths (95% CI) | | | | | | | |
| --- | --- | --- | --- | --- | --- | --- | --- | --- |
|  | IHD | | Stroke | | ALRI | | COPD | |
|  | Main Model | IHME | Main Model | IHME | Main Model | IHME | Main Model | IHME |
| 2010 | 1,359 (835–1,854) | 3,967 (2,757–5,282) | 1,009 (136–1,847) | 5,451 (3,978–7,108) | 676 (359–978) | 1,792 (220–3,321) | 1,315 (887–1,709) | 1,744 (1,272–2,333) |
| 2011 | 1,381 (848–1,885) | 4,189 (2,961–5,540) | 955 (128–1,748) | 5,567 (4,113–7,223) | 766 (406–1,109) | 2,008 (249–3,701) | 1,271(858–1,654) | 1,831 (1,345–2,438) |
| 2012 | 1,329 (813–1,819) | 4,511 (3,220–5,907) | 868 (116–1,593) | 5,871 (4,332–7,589) | 825 (436–1,197) | 2,294 (287–4,194) | 1,292 (868–1,687) | 1,975 (1,452–2,633) |
| 2013 | 1,352 (830–1,846) | 4,812 (3,441–6,302) | 950 (128–1,739) | 6,104 (4,476–7,863) | 949 (503–1,374) | 2,557 (314–4,661) | 1,283 (865–1,670) | 2,100 (1,541–2,824) |
| 2014 | 1,446 (888–1,975) | 5,064 (3,579–6,628) | 918 (123–1,680) | 6,307 (4,583–8,142) | 1,074 (569–1,554) | 2,824 (347–5,157) | 1,318 (889–1,715) | 2,221 (1,621–2,995) |
| 2015 | 1,482 (909–2,025) | 5,280 (3,717–6,991) | 911 (122–1,669) | 6,508 (4,689–8,498) | 1,320 (699–1,910) | 3,098 (383–5,670) | 1,393 (939–1,813) | 2,326 (1,672–3,180) |
| 2016 | 1,468 (901–2,005) | 5,420 (3,834–7,158) | 871 (117–1,596) | 6,573 (4,755–8,639) | 1,459 (773–2,113) | 3,306 (427–5,992) | 1,288 (868–1,676) | 2,379 (1,696–3,267) |
| 2017 | 1,370 (840–1,874) | 5,528 (3,790–7,335) | 814 (109–1,491) | 6,640 (4,691–8,862) | 1,647 (871–2,387) | 3,506 (452–6,371) | 1,203 (810–1,568) | 2,431 (1,681–3,427) |
| 2018 | 1,277 (781–1,750) | 5,693 (3,753–7,675) | 751 (101–1,379) | 6,748 (4,585–9,230) | 1,805 (953–2,621) | 3,688 (455–6,778) | 1,069 (717–1,398) | 2,502 (1,649–3,585) |
| 2019 | 1,230 (753–1,685) | 5,682 (3,511–7,926) | 717 (96–1,316) | 6,688 (4,287–9,554) | 1,880 (994–2,727) | 3,739 (453–7,067) | 1,026 (689–1,340) | 2,535 (1,544–3,768) |
| Total | 13,696 (8,398–18,718) | 50,147 (34,563–66,744) | 8,765 (1,176–16,059) | 62,458 (44,489–82,709) | 12,400 (6,562–17,969) | 28,812 (3,589–52,912) | 12,459 (8,390–16,231) | 22,045 (15,474–30,449) |

**Abbreviations:** CI, confidence interval; IHD, ischemic heart disease; ALRI, acute lower respiratory infections; COPD, chronic obstructive pulmonary disease;

**Note:** The estimates for excess deaths in this table from IHME are based on the Global Burden of Disease (GBD) 2021 data (https://vizhub.healthdata.org/).
